# Severe hyposmia distinguishes neuropathologically confirmed dementia with Lewy bodies from Alzheimer’s disease dementia

**DOI:** 10.1371/journal.pone.0231720

**Published:** 2020-04-22

**Authors:** Thomas G. Beach, Charles H. Adler, Nan Zhang, Geidy E. Serrano, Lucia I. Sue, Erika Driver-Dunckley, Shayamal H. Mehta, Edouard E. Zamrini, Marwan N. Sabbagh, Holly A. Shill, Christine M. Belden, David R. Shprecher, Richard J. Caselli, Eric M. Reiman, Kathryn J. Davis, Kathy E. Long, Lisa R. Nicholson, Anthony J. Intorcia, Michael J. Glass, Jessica E. Walker, Michael M. Callan, Javon C. Oliver, Richard Arce, Richard C. Gerkin

**Affiliations:** 1 Banner Sun Health Research Institute, Sun City, Arizona, United States of America; 2 Department of Neurology, Mayo Clinic, Scottsdale, Arizona, United States of America; 3 Department of Biostatistics, Mayo Clinic, Scottsdale, Arizona, United States of America; 4 Cleveland Clinic Lou Ruvo Center for Brain Health, Las Vegas, Nevada, United States of America; 5 Barrow Neurological Institute, Phoenix, Arizona, United States of America; 6 Banner Alzheimer’s Institute, Phoenix, Arizona, United States of America; 7 School of Life Sciences, Arizona State University, Tempe, Arizona, United States of America; Nathan S Kline Institute, UNITED STATES

## Abstract

Many subjects with neuropathologically-confirmed dementia with Lewy bodies (DLB) are never diagnosed during life, instead being categorized as Alzheimer’s disease dementia (ADD) or unspecified dementia. Unrecognized DLB therefore is a critical impediment to clinical studies and treatment trials of both ADD and DLB. There are studies that suggest that olfactory function tests may be able to distinguish DLB from ADD, but few of these had neuropathological confirmation of diagnosis. We compared University of Pennsylvania Smell Identification Test (UPSIT) results in 257 subjects that went on to autopsy and neuropathological examination. Consensus clinicopathological diagnostic criteria were used to define ADD and DLB, as well as Parkinson’s disease with dementia (PDD), with (PDD+AD) or without (PDD-AD) concurrent AD; a group with ADD and Lewy body disease (LBD) not meeting criteria for DLB (ADLB) and a clinically normal control group were also included. The subjects with DLB, PDD+AD and PDD-AD all had lower (one-way ANOVA p < 0.0001, pairwise Bonferroni p < 0.05) first and mean UPSIT scores than the ADD, ADLB or control groups. For DLB subjects with first and mean UPSIT scores less than 20 and 17, respectively, Firth logistic regression analysis, adjusted for age, gender and mean MMSE score, conferred statistically significant odds ratios of 17.5 and 18.0 for the diagnosis, vs ADD. For other group comparisons (PDD+AD and PDD-AD vs ADD) and UPSIT cutoffs of 17, the same analyses resulted in odds ratios ranging from 16.3 to 31.6 (p < 0.0001). To our knowledge, this is the largest study to date comparing olfactory function in subjects with neuropathologically-confirmed LBD and ADD. Olfactory function testing may be a convenient and inexpensive strategy for enriching dementia studies or clinical trials with DLB subjects, or conversely, reducing the inclusion of DLB subjects in ADD studies or trials.

## Introduction

Dementia due to AD (ADD) is often associated with comorbid brain disease that may affect clinical presentation, rate of cognitive decline, and response to therapeutic agents [1–22]. Additional concurrent pathology could be unresponsive to therapies directed at the “primary” pathology. It is apparent then, that clinical trials for ADD could suffer from decreased effect size if this were true.

The most common comorbidity in ADD is Lewy body disease. Slightly more than one-half or more of all those meeting clinicopathological ADD diagnostic criteria also have α-synuclein pathology [9,23–25] with morphological features similar to Parkinson’s disease (PD). This is broadly termed “Lewy body disease” (LBD). Similarly, about one-half of those with dementia and PD (PDD) [26–38] and three-quarters or more of those with dementia with Lewy bodies (DLB), have clinically significant AD histopathology [39–42]. In the majority of subjects with ADD and DLB (ADD/DLB), the typical clinical signs and symptoms of DLB [43,44] are absent and thus this co-existence is recognized only at autopsy [22,45–47]. This clinical inability to separate ADD from DLB hampers clinical trials for both conditions. Several autopsy-validated studies have indicated that cognitive decline is faster in elderly subjects dying with ADD who also have LBD [3,22,48–51], and disease duration has been reported to be shorter in those with coexistent ADD and DLB [40,52]. There is therefore a critical need for better clinical differentiation of these two conditions.

There are numerous published clinical studies that suggest that olfactory function tests may be useful in differentiating amongst cerebrovascular and neurodegenerative disorders [53–64] and, in particular, in distinguishing DLB from ADD [65–70], but the studies with later neuropathological establishment of the specific molecular pathology are the most informative. Possibly the first such study, done by Oxford University [71], investigated the neuropathological correlates of anosmia in subjects with dementia. Anosmia was defined on the basis of being able or unable to detect the scent of lavender oil. Seventeen subjects had neuropathological DLB, defined as the concurrent presence of Lewy bodies in both the substantia nigra and cingulate gyrus. Sixteen of these had concurrent ADD while another 43 subjects had ADD alone, defined as probable or definite CERAD AD [72], without LBD. Anosmia was significantly (p = 0.029) more common in DLB (41%) than in ADD (16%).

A similar study [73] from the University of Southern California defined anosmia as the inability to detect the odor of N-butyl alcohol, finding anosmia in 47% of those with the Lewy body variant (LBV, n = 17) of AD versus 22% of those with AD alone (n = 89). This proportional difference was highly significant (p = 0.0004). The diagnosis of LBV was defined as the presence of Lewy bodies in both brainstem and cerebral cortex while ADD was defined as CERAD probable or definite AD. The independent odds ratio for anosmia as a predictor of LBV was 5.4, vs 7.3 for visual hallucinations.

In a study of a mixed group of non-demented and demented subjects with and without parkinsonism from the Rush Memory and Aging Project [74], lower scores on the Brief Smell Identification Test were significant predictors of limbic and neocortical LBD stages (9 and 13 subjects, respectively). The presence of any Lewy bodies accounted for 15.4% of test variance, as compared to 4.1% due to a composite measure of AD histopathology.

Incidental Lewy body disease (ILBD) refers to the presence of LBD in asymptomatic elderly people and is likely to be a prodromal stage of PD or DLB as striatal dopaminergic markers are halfway between asymptomatic elderly people without LBD and clinically-manifest PD [75–78]. One neuropathologically-informed study has reported that olfactory function in subjects with ILBD is also halfway between PD and asymptomatic elderly people without LBD, suggesting that hyposmia may be useful as a prodromal marker [79]. Another prior study found an OR of 11.0 for the prediction of ILBD in those amongst the lowest tertile of olfactory function [80]. Supporting the possible usefulness of hyposmia as a prodromal biomarker are the findings that it is present in some clinically normal GBA and LRRK2 mutation carriers [81,82], is common in idiopathic REM sleep behavior disorder (iRBD) [83–85], is a significant and independent predictor of phenoconversion from iRBD to parkinsonism or dementia [86] and is associated with decreased striatal dopamine transporter imaging [87,88]. A likely causative factor underlying the impairment of olfaction in LBD is the near-universal occurrence of early-stage α-synuclein pathology within the olfactory bulb [89–92].

In this study we sought to determine the diagnostic utility of hyposmia as a diagnostic predictor of neuropathologically-identified DLB with comorbid ADD, as compared with ADD alone, using the largest set to date of neuropathologically-examined subjects. Furthermore, we did similar analyses on the diagnostic utility of hyposmia for distinguishing Parkinson’s disease with dementia from ADD alone.

## Materials and methods

### Subject selection

Subjects were selected by database searches of the Arizona Study of Aging and Neurodegenerative Disorders (AZSAND)/ Banner Sun Health Research Institute Brain and Body Donation Program (www.brainandbodydonationprogram.org) [93], a subset of whom were also enrolled in the National Institute on Aging Arizona Alzheimer’s Disease Core Center. Search criteria specified that subjects died with dementia, one or more completed University of Pennsylvania Smell Identification Tests (UPSIT) accompanied by Mini Mental State Examinations (MMSE), assessments of the presence or absence of parkinsonism and visual hallucinations, and a full neuropathological examination after death. Selected subjects met “intermediate” or “high” National Institute on Aging-Reagan Institute (NIA-RI) clinicopathological criteria [94] for ADD, with or without also meeting “intermediate” or “high” clinicopathological criteria for DLB [43,44], or alternatively, for groups with Parkinson’s disease dementia or Alzheimer’s disease with Lewy body pathology (ADLB) [91], the latter defined as having pathologically-confirmed CNS LBD but not meeting DLB pathology distribution and density thresholds. Briefly, intermediate and high NIA-RI criteria stipulate Braak neurofibrillary stages III or IV versus V and VI, respectively. DLB intermediate and high criteria are based on comparison of Lewy body pathology stage with AD pathology stage; when AD pathology stage is high, only the neocortical Lewy body stage qualifies for DLB, while when AD pathology stage is intermediate, either a limbic or neocortical Lewy body stage qualifies for DLB. Parkinson’s disease with dementia (PDD) is defined as meeting clinicopathological diagnostic criteria for PD as well as clinical criteria for dementia, and is further subdivided by the presence (PDD+AD) or absence (PDD-AD) of intermediate or high NIA-RI criteria AD pathology. ADLB is defined as intermediate or high NIA-RI AD criteria together with any Lewy body stage not qualifying for DLB, which can be either limbic or brainstem stages. A control subject group, without clinical parkinsonism or dementia, and without α-synuclein pathology at autopsy, was also included. For all subjects, most other major neuropathological disorders were excluded; this included subjects with progressive supranuclear palsy, multiple system atrophy and corticobasal degeneration. As mean UPSIT did not differ between ADD and the neuropathologically-defined ADLB (n = 30) and AD-VaD (n = 25) groups, these were grouped for the primary analyses. Cases with DLB in the absence of intermediate or high NIA-RI status were not included in statistical analyses in this study as there were only 4 cases with UPSIT data available.

### Subject characterization

Most subjects had serial standardized research cognitive evaluations, done by teams of nurses, medical assistants, behavioral neurologists, movement disorders neurologists, neuropsychologists and psychometrists using standardized research-quality assessment batteries [93], including the Mini Mental State Examination (MMSE), National Alzheimer’s Coordinating Center (NACC) Uniform Data Set (UDS) and the Unified Parkinson’s Disease Rating Scale (UPDRS). Subjects had olfactory testing with the University of Pennsylvania Smell Identification Test (UPSIT) [95–97] every third year on average. The presence or absence of DLB core clinical features [43,44], including the presence or absence of parkinsonism, visual hallucinations, fluctuations in attention or cognition and clinical history consistent with REM sleep behavior disorder (RBD), were recorded for each subject at each visit; to assist with the latter, the Mayo Sleep Questionnaire [98–101] was administered. The presence or absence of parkinsonism and visual hallucinations were noted for 103 and 88 (respectively) of the 125 total ADD, ADLB and ADD/DLB subjects, by formal examination as part of the Unified Parkinson’s Disease Rating Scale (UPDRS) and/or as part of the Uniform Data Set (UDS) of the National Alzheimer’s Coordinating Center (NACC). For those that did not have either UPDRS or UDS data available, the presence of parkinsonism and visual hallucinations was additionally noted by review of private medical records. These determinations were made within the same year (matched year) as the first UPSIT administration, for comparison with first UPSIT as a diagnostic predictor, while for comparison with mean UPSIT score, the cumulative recorded presence of visual hallucinations and parkinsonism, at any timepoint within the clinical observation period was used.

All subjects received identical neuropathological examinations, including summary regional brain density measures for total amyloid plaques, neurofibrillary tangles, Lewy body pathology regional and summary density scoring, and staging using the Unified Staging System for Lewy Body Disorders [91], as well as assignment of CERAD neuritic plaque density and Braak neurofibrillary stage, as described previously [93].

The corresponding author had access to personally-identifying information for subjects in this study.

### Statistical analysis

Demographic and post-mortem characteristics were analyzed using one-way analysis of variance (ANOVA), Chi-square tests and unpaired t-tests as appropriate. Receiver-operator characteristics analysis was implemented for first and mean UPSIT scores to separately predict the diagnosis of DLB, ADLB, PDD+AD and PDD-AD vs ADD. Youden index was used as the criteria to choose the optimum cut-off point for UPSIT scores. In the analyses comparing ADD/DLB and ADLB with ADD, sensitivity, specificity, and accuracy for predicting the presence of Lewy body disease, based on UPSIT cutoff scores vs the presence or absence of visual hallucinations and parkinsonism, were further calculated. Firth logistic regression models adjusted for age, gender and corresponding MMSE scores were used to estimate odds ratios for different predictors and areas under the curve (AUC) for each model. The AUCs for the models were compared using Delong’s method [102].

## Results and discussion

Clinical, demographic and neuropathological characteristics of the compared groups (total n = 287) are shown in Table 1. Of the disease groups, the PDD+AD and PDD-AD groups were the youngest, had the highest final UPDRS scores, the highest final MMSE scores, the lowest plaque and tangle scores and the highest Lewy pathology scores. The ADD/DLB, ADLB, PDD+AD and PDD groups all had higher proportions of men. The ADLB group had the lowest final MMSE score. As expected, the control group was significantly different from the dementia groups in all clinical and neuropathological measures. Neuropathologically, the ADD and ADD/DLB groups were not different in their density scores or stages for amyloid plaques (total plaques), neuritic plaques and neurofibrillary tangles.

**Table 1:** Clinical and neuropathological characteristics of study subjects.

	ADD (n = 66)	ADD/DLB (n = 29)	ADLB N = 30)	PDD+AD (n = 21)	PDD-AD (n = 27)	Control (n = 84)	p-value
**Age (yrs)**^1^	88.2 (6.9)	85.1 (7.4)	87.0 (7.6)	83.2 (4.7)	78.2 (7.5)	86.7 (6.9)	< 0.0001
**Gender (M/F)**	50/46	22/7	23/7	13/8	22/5	38/46	< 0.01
**Last MMSE score**^2^	18.3 (8.3)	17.1 (7.0)	14.6 (8.3)	20.0 (5.5)	21.1 (5.5)	28.3 (1.4)	< 0.0001
**Last UPDRS Score**^3^	16.5 (15.9)	23.7 (20.0)	15.8 (13.8)	41.8 (16.6)	43.2 (17.1)	8.5 (8.8)	< 0.0001
**Plaque Score**^4^	12.98 (2.42)	11.48 (3.85)	14.0 (0.99)	11.3 (3.7)	1.44 (2.7)	5.21 (5.68)	< 0.0001
**Tangle Score**^5^	10.77 (3.45)	9.24 (3.86)	12.4 (2.7)	6.5 (2.3)	5.3 (2.8)	4.82 (2.51)	< 0.0001
**LB Score**^6^	3.28 (6.91)	21.19 (5.52)	10.8 (8.8)	32.0 (6.6)	27.6 (5.1)	0	< 0.0001

Means and standard deviations are shown. ADD = Alzheimer’s disease dementia; DLB = dementia with Lewy bodies; MMSE = last Mini Mental State Examination score; UPDRS = last Unified Parkinson’s Disease Rating Scale motor score (part 3 score, off medications); Plaque Score and Tangle Score = summary regional brain density scores with maximum scores of 15. LB Score = summary regional brain Lewy-type synucleinopathy density score with a maximum score of 40. All values in the p-value column are for one-way analysis of variance except for gender, where chi-square analysis was done.

^1^. Post-hoc paired Bonferroni significance testing significant (p<0.05) for PDD+AD and PDD-AD vs all other groups.

^2^. N = 29 for ADLB; Post-hoc paired Bonferroni significance testing significant (p<0.05) for all groups vs control and for ADLB vs PDD+AD and PDD-AD.

^3^. N = 94, 27, 29, 15, 21 and 82 for ADD, ADD/DLB, ADLB, PDD+AD, PDD-AD and control, respectively. Post-hoc paired Bonferroni significance testing significant (p<0.05) for all groups vs control except ADD vs control, and significant for ADD, ADLB and ADD/DLB vs PDD+AD and PDD-AD.

^4^. Post-hoc paired Bonferroni testing significant (p < 0.05) for all groups vs control and for ADD and for all groups vs PDD-AD.

^5^. N = 95 for ADD; Post-hoc paired Bonferroni testing significant (p < 0.05) for PDD+AD, PDD-AD and control vs ADD and ADD/DLB.

^6^. N = 92, 26, 28, 20 for ADD, ADD/DLB, ADLB, PDD+AD respectively; Post-hoc paired Bonferroni testing significant (p < 0.05) for control and ADD vs all groups and for ADLB vs ADD/DLB, PDD+AD and PDD-AD.

**Fig 1:**
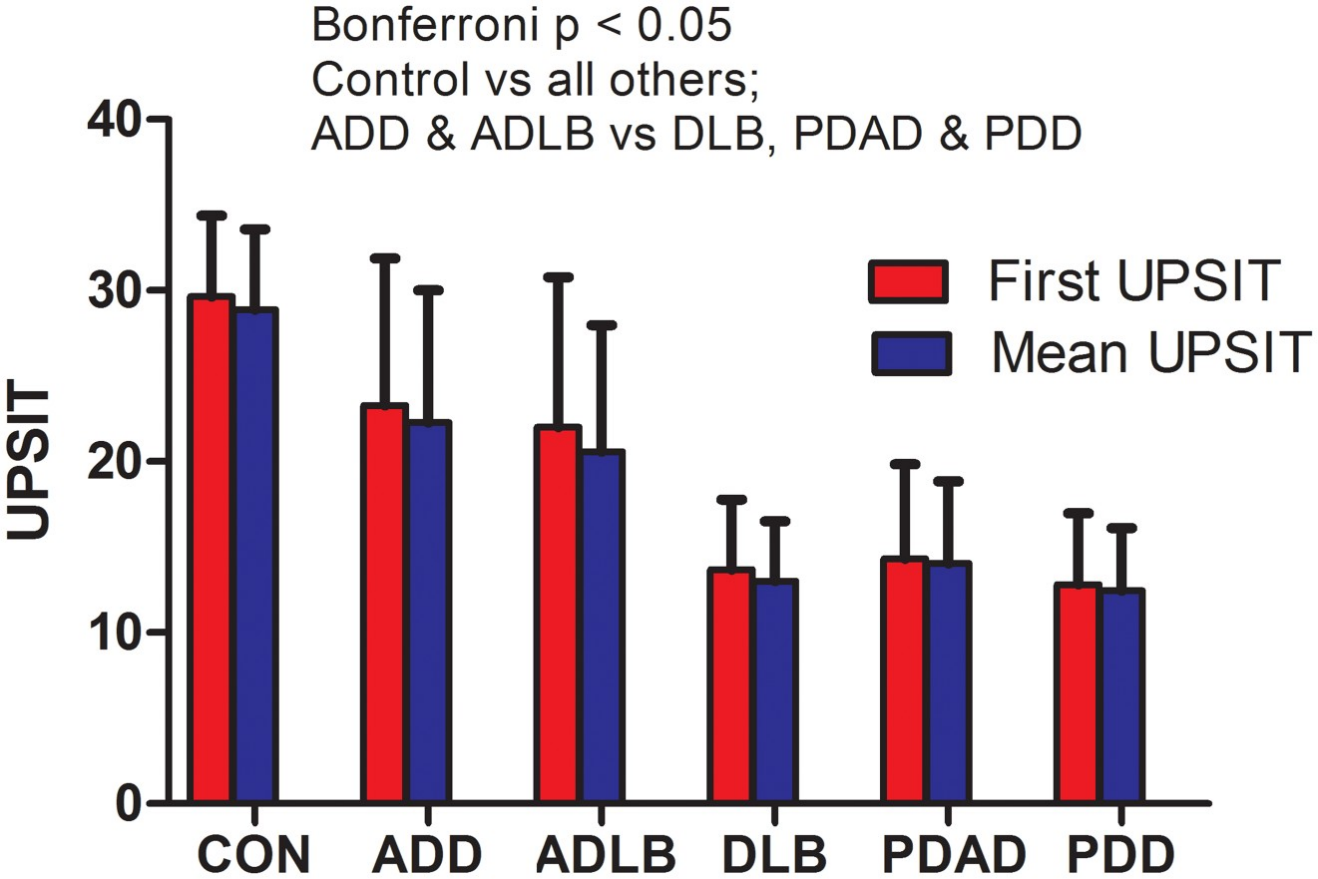
First and mean UPSIT scores in the diagnostic groups. Both first UPSIT and mean UPSIT scores are significantly different between groups (ANOVA, p < 0.001). For both first and mean UPSIT scores, the control group scores are significantly higher than all other groups, and both the ADD and ADLB groups have mean scores that are significantly higher than the ADD/DLB, PDD+AD (PDAD on the graph) and PDD-AD (PDD on the graph) groups (Bonferroni p < 0.05). First and mean UPSIT scores were not significantly different within diagnostic groups. Error bars = standard deviation.

Comparison of UPSIT scores, including the first UPSIT score and the mean of all UPSIT scores, showed that all groups with Lewy body pathology, except the ADLB group, had significantly lower UPSIT scores than the non-Lewy body pathology groups (Fig 1). The mean UPSIT score for the 4 DLB subjects without ADD was 12.8 (not shown on graph).

Control subjects had significantly more UPSITs (mean 1.9, range 1–4) than ADD (mean 1.6, range 1–4), ADD/DLB (mean 1.35, range 1–3) ADLB (mean 1.52; range 1–3), PDD+AD (1.33; 1–3) or PDD-AD (1.37, range 1–2) groups but this did not differ between the diseased groups. Logistic regression analysis of the combined disease groups (n = 163 after exclusion of cases with incomplete neuropathology scores) found a unitary increase in the brain regional sum of α-synuclein pathology score (maximum score of 40) was significantly associated with a mean UPSIT score less than the median of all cases (OR 1.12, 95% CI 1.08–1.17, p < 0.000001). Higher regional brain scores for amyloid plaque density score as well as higher age at death were not significant predictors, while higher neurofibrillary tangle density scores as well as lower last MMSE test scores independently approached the significance level (p = 0.08 and p = 0.07, respectively).

**Fig 2:**
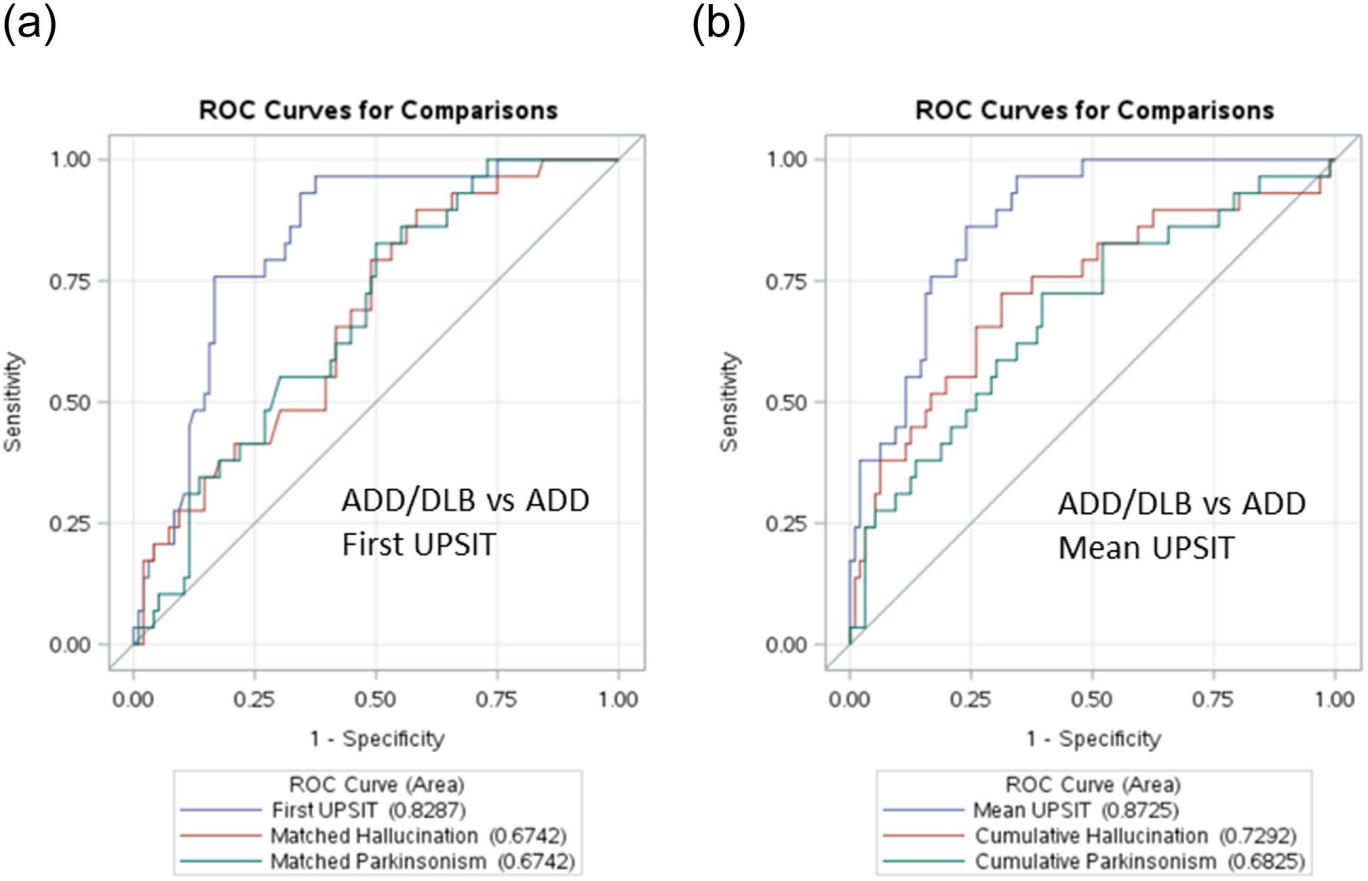
Comparison of ROC curves for the discrimination of ADD/DLB vs ADD using a) using first UPSIT scores and b) using mean UPSIT scores, with those using presence or absence of visual hallucinations and parkinsonism within the same year of observation.

Logistic regression analysis and receiver-operator characteristics (ROC) indicated that the UPSIT cutoff scores giving the greatest accuracy for separating ADD from ADD/DLB were 19 or less for the first UPSIT (median of ADD/DLB and ADD first UPSIT scores was 20) and 17 for the mean UPSIT. Using these cutoffs, a first UPSIT score less than 20 gave an odds ratio (OR) of 17.5 for a diagnosis of ADD/DLB, while for mean UPSIT, a score less than 17 resulted in an OR of 18.0 for the diagnosis (Table 2). These ORs were considerably greater than those derived from the presence or absence of the two most common DLB core clinical features, visual hallucinations and parkinsonism (Table 2, Fig 2a and 2b), and were highly significant (p < 0.0001) whereas only the OR for cumulatively-observed hallucinations was significant (OR 3.3; p = 0.01). Similarly, the area under the curves (AUC) were significantly greater for first and mean UPSIT as compared with those for matched-year or cumulative hallucinations and parkinsonism.

**Table 2:** Comparison of first UPSIT score and mean of all UPSIT scores with visual hallucinations and parkinsonism as predictors of ADD/DLB vs ADD.

Predictor	Sensitivity	Specificity	Accuracy	Odds Ratio (95% CI), p-value	AUC	P-value
**First UPSIT**^1^	93.1%	64.6%	71.2%	17.5 (5.1, 91.6) < .0001	82.9%	0.2419^3^
**Matched Year hallucinations**^1^	17.2%	96.9%	78.4%	4.4 (0.9, 25.0) 0.0905	67.4%	0.0012^4^
**Matched Year parkinsonism**^1^	31.0%	77.1%	66.4%	1.7 (0.7, 4.3) 0.2648	67.4%	0.0006^4^
**Mean UPSIT**^2^	86.2%	71.9%	75.2%	18.0 (6.0, 66.8) < .0001	87.2%	0.2419^3^
**Cumulative hallucinations**^2^	51.7%	76.0%	70.4%	3.3 (1.4, 8.4) 0.0106	72.9%	0.008^4^
**Cumulative parkinsonism**^2^	65.5%	45.8%	50.4%	1.6 (0.7, 3.9) 0.3001	68.2%	0.0007^4^

“Matched” indicates that determinations of the presence or absence of hallucinations and parkinsonism were done close to the same year as the first UPSIT examination.

^1^. Adjusted for matched year MMSE and age at first UPSIT.

^2^. Adjusted for mean MMSE and age at death.

^3^. P-value comparing AUCs for first and mean UPSIT.

^4^. P-value comparing AUC with first UPSIT.

^5^. P-value comparing AUC with mean UPSIT.

**Fig 3:**
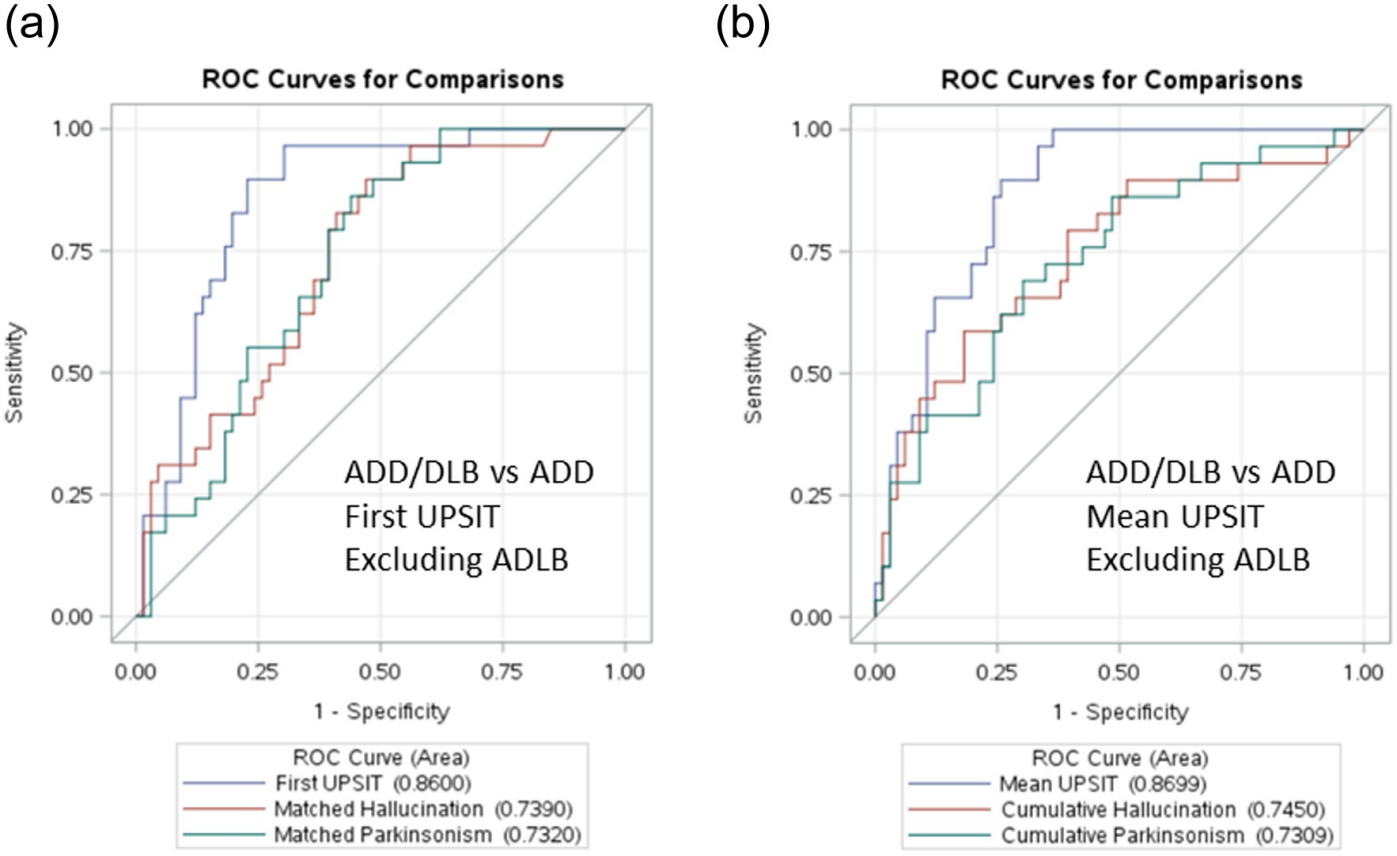
Comparison of ROC curves after exclusion of 30 ADLB subjects from the ADD group. a) using first UPSIT scores and b) using mean UPSIT scores.

**Fig 4:**
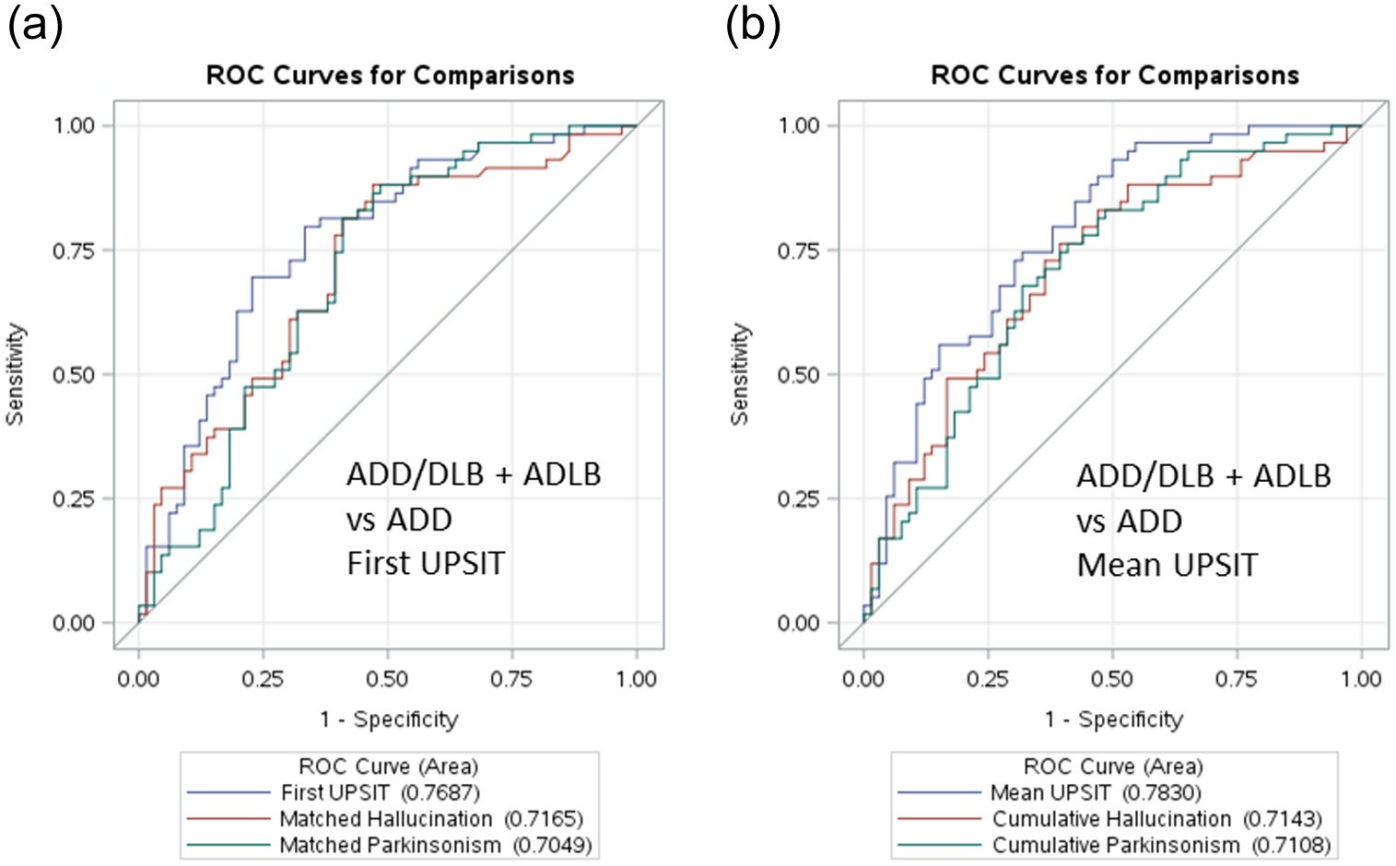
Comparison of ROC curves after combining the ADLB subjects with the ADD/DLB subjects. a) using first UPSIT scores and b) using mean UPSIT scores.

To determine whether the inclusion of ADLB subjects with the ADD group affected the primary analysis of ADD/DLB vs ADD, logistic regression analysis and ROC curves were used to compare the ADD and ADD/DLB groups after exclusion of the 30 subjects with ADLB, and to compare the ADD group with a composite ADLB-DLB group. The results after exclusion of the ADLB group were very similar to those obtained with the ADD and ADLB groups together (Fig 3a and 3b). All comparisons that were statistically significant in the primary analysis were also significant after exclusion of the ADLB subjects. A first UPSIT cutoff score of 19 gave an OR of 28.3 for separating ADD from ADD/DLB while a mean UPSIT cutoff of 20 gave an OR of 24.4 for predicting ADD/DLB (both with p < 0.0001). The results with ADLB and ADD/DLB subjects grouped together and compared with the ADD group showed much less predictive power for both first and mean UPSIT scores (Fig 4a and 4b).

**Fig 5:**
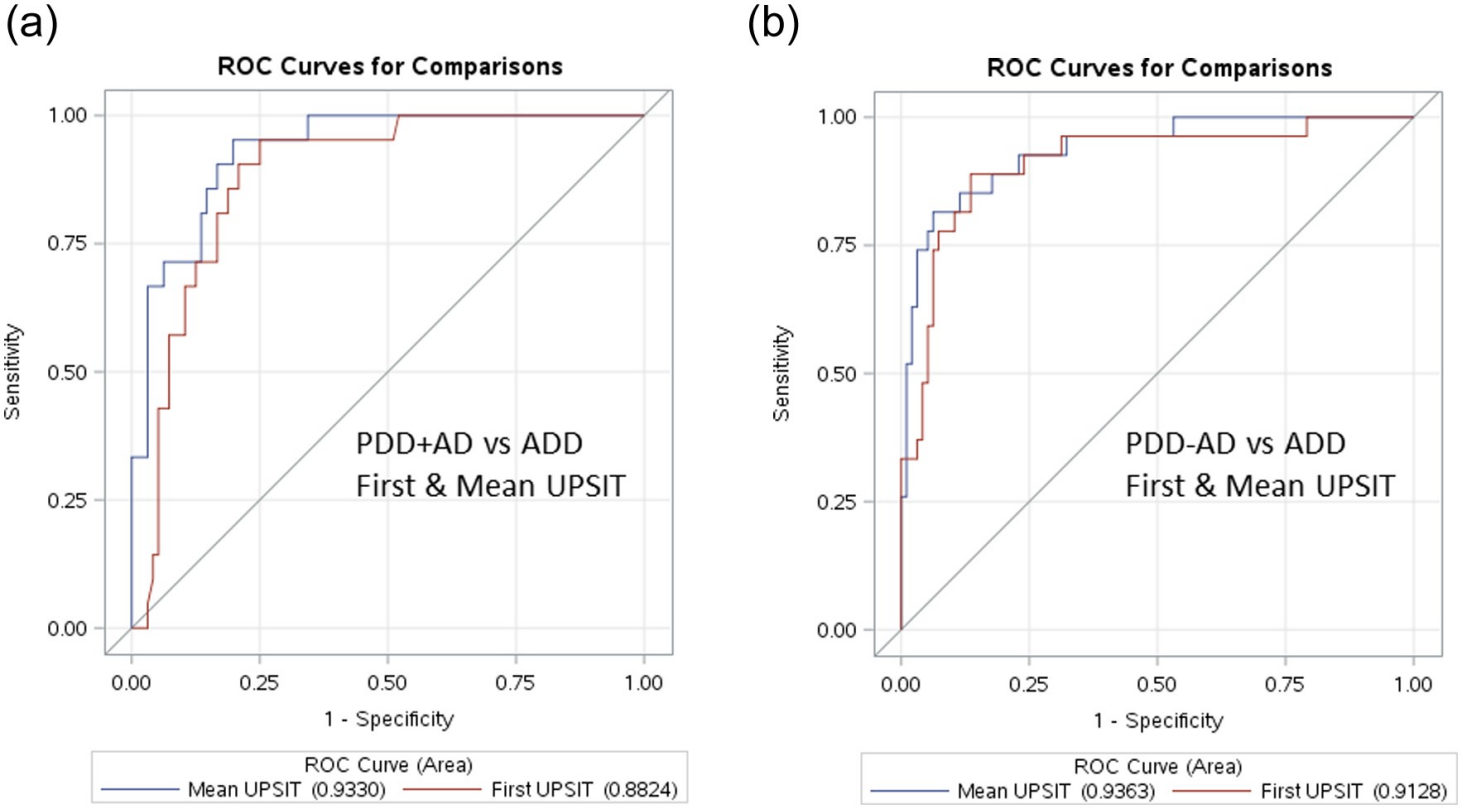
ROC curves for the discrimination of a) PDD+AD vs ADD subjects and b) PDD-AD, using first and mean UPSIT scores.

With the same analyses applied to the discrimination of PDD+AD and PDD-AD from ADD (Fig 5a and 5b), odds ratios were of similar magnitudes with similar UPSIT cutoffs. For PDD+AD vs ADD (Fig 5a), a first UPSIT cutoff score of 17 gave an OR of 20.3 for separating PD+AD from ADD, while a mean UPSIT cutoff of 17 gave an OR of 31.6 this distinction (both with p < 0.0001). For PDD-AD vs ADD (Fig 5a), a first UPSIT cutoff score of 17 gave an OR of 16.3 for separating PDD-AD from ADD, while a mean UPSIT cutoff of 17 gave an OR of 25.7 for this distinction (both with p < 0.0001).

The results of this study show that the presence of diagnostically significant brain loads of α-synuclein pathology have a pronounced effect on olfaction that is much greater than that conferred by ADD pathology, and that this might be exploited to make the clinical differentiation between such subjects, even in the absence of other clinical features. Why ADLB subjects would have relatively preserved olfaction compared to ADD/DLB subjects was initially puzzling to us, as both have severe α-synuclein pathology in the olfactory bulb (median score of 4/4 in both groups in this study). However, the frontal cortex and amygdala also contribute to the subjective sense of smell [103], and for both of these areas, α-synuclein pathology is significantly more severe in ADD/DLB than in ADLB with median amygdala scores of 4/4/ vs 3/4 and median frontal cortex scores of 1/4/ vs 0/4, respectively (p < 0.001, Mann-Whitney U-tests).

ADD and DLB often co-exist unknown to clinicians, and as this comorbidity may affect the presentation, rate of cognitive decline, and response to therapeutic agents [1–22], clinical trials for both conditions may be impaired. The most common comorbidity in ADD is LBD, affecting somewhat more than one-half or more of all those meeting clinicopathological ADD diagnostic criteria [9,23–25]. Dementia with Lewy bodies has greater α-synuclein pathology than ADLB and therefore may be more resistant to therapeutic agents targeting AD molecular lesions. In the majority of subjects with ADD and DLB, the typical clinical signs and symptoms of DLB [43,44] are absent and thus this co-existence is recognized only at autopsy [22,45–47]. Disease duration has been reported to be shorter in those with coexistent ADD and DLB [40,52]. There is therefore a critical need for better clinical differentiation of these two conditions.

Definitive laboratory-based biomarkers for DLB are not yet available. Molecular imaging of striatal dopamine receptors, and myocardial scintigraphy with [123] meta-iodobenzylguanidine (MIBG) have both been used as diagnostic adjuncts for DLB [104,105] with promising but not yet definitive results from small autopsy-confirmed studies [106,107]. Dopaminergic imaging may be less helpful in DLB as compared to PD, due to less consistent degeneration of nigrostriatal dopaminergic neuronal and nerve terminals [108–112]. Biofluids and PET imaging approaches have so far been unsuccessful in providing the required accuracy for identifying LBD [113–115]. Simulation studies have suggested that cortical biopsy [116–119] would have high sensitivity and specificity for DLB, and usage of needle cores rather than open biopsy may reduce morbidity to acceptable levels [116]. Biopsy of the peripheral nervous system [120], particularly the submandibular gland [121–124], also shows promise for diagnosing DLB. Autopsy studies have suggested that biopsy of the olfactory bulb would identify more than 90% of all subjects with LBD [90]. Better clinical diagnostic methods for DLB are critically needed, as improved sensitivity in the clinical identification of DLB would greatly assist recruitment for clinical trials and would allow exclusion or stratification of DLB subjects within ADD clinical trials.

Numerous studies have indicated the potential for olfactory function tests to distinguish different cerebrovascular and neurodegenerative disorders [53–64] and, in particular, to distinguish PD and DLB from ADD [65–70], but the great majority of these studies lack certainty due the reliance on a clinical diagnosis as gold standard. Several studies with neuropathological confirmation of LBD have suggested that loss of olfactory function may be more pronounced in DLB but these have been limited by small subject numbers [71–74].

As the first and mean UPSIT scores were not significantly different, it seems probable that hyposmia is a relatively early clinical occurrence in ADD/DLB, and hence smell testing could be helpful in the identification of prodromal DLB. Support for this possibility comes from studies of incidental Lewy body disease (ILBD), defined as the presence of LBD in asymptomatic elderly people. ILBD is a probable prodromal stage of PD or DLB as dopaminergic markers are halfway between asymptomatic elderly people without LBD and clinically-manifest PD [75–78]. Our group has previously reported that olfactory function in subjects with ILBD is also halfway between PD and asymptomatic elderly people without LBD [79]; another clinicopathological study found an OR of 11.0 for hyposmia in the prediction of ILBD, using as a cutoff the lowest tertile of olfactory function [80], and these postmortem studies have been further confirmed by the in vivo association of hyposmia with decreased striatal dopamine transporter imaging [87,88]. Additional support comes from reports of hyposmia in some clinically normal GBA and LRRK2 mutation carriers [81,82] and in idiopathic REM sleep behavior disorder (iRBD) [83–86].

## Conclusions

In this study we sought to determine the diagnostic utility of hyposmia as a diagnostic predictor of neuropathologically-identified ADD/DLB, using considerably larger subject numbers than previous studies. Our results confirm those of the prior studies, where subjects with ADD/DLB have been repeatedly found to have worse olfactory function than ADD. The odds ratios for ROC-determined first and mean UPSIT score cutoffs, 17.5 and 18.0, respectively, were surprisingly stronger than the ORs for both visual hallucinations and parkinsonism (1.7–4.4), two of the key core clinical DLB features. These figures suggest that olfactory testing should be considered as a core clinical feature of DLB and could potentially be of great assistance in the clinical separation of ADD and DLB, allowing stratification of clinical trial subjects. Larger neuropathologically-examined subject numbers would help to confirm the results of the present study but if results from the prior three neuropathologically-confirmed studies are added to this, there are 137 LBD cases and 365 controls (ADD or normal controls), all with the same general finding of much lower olfactory test scores in the LBD groups. We cannot be sure, due to insufficient numbers of pure DLB cases in our study, that olfactory testing might be equally useful for the separation of pure DLB from ADD, but the low mean UPSIT score of the 4 cases in the present study is consistent with that observed for the mixed ADD/DLB cases.

## Supporting information

S1 Data (XLSX)
